# The effects of physical activity on sleep architecture and mood in naturalistic environments

**DOI:** 10.1038/s41598-024-56332-7

**Published:** 2024-03-07

**Authors:** Kennedy Zapalac, Melissa Miller, Frances A. Champagne, David M. Schnyer, Benjamin Baird

**Affiliations:** Department of Psychology, The University of Texas at Austin, 108 E Dean Keeton St, Austin, TX 78712 USA

**Keywords:** Sleep, Physical activity, Exercise, Sleep architecture, REM sleep, REM latency, Experience sampling, Wearable devices, Circadian rhythms and sleep, Psychology

## Abstract

Physical activity has been found to alter sleep architecture, but these effects have been studied predominantly in the laboratory and the generalizability of these findings to naturalistic environments and longer time intervals, as well as their psychological effects, have not been evaluated. Recent technological advancements in wearable devices have made it possible to capture detailed measures of sleep outside the lab, including timing of specific sleep stages. In the current study, we utilized photoplethysmography coupled with accelerometers and smartphone ambulatory assessment to collect daily measurements of sleep, physical activity and mood in a sample of N = 82 over multi-month data collection intervals. We found a robust inverse relationship between sedentary behavior and physical activity and sleep architecture: both low-intensity and moderate-to-vigorous physical activity were associated with increased NREM sleep and decreased REM sleep, as well as a longer REM latency, while higher levels of sedentary behavior showed the opposite pattern. A decreased REM/NREM ratio and increased REM latency were in turn associated with improved wellbeing, including increased energy, reduced stress and enhanced perceived restfulness of sleep. Our results suggest that physical activity and sleep account for unique variance in a person’s mood, suggesting that these effects are at least partially independent.

## Introduction

Physical activity and sleep are two major public health targets due to the numerous physical, cognitive, and mental health benefits that are conferred by each of these behaviors^1^. Both adequate sleep and sufficient physical activity have been shown to reduce the risk of all-cause mortality and prevent or reduce the risk of mortality from chronic conditions, including hypertension, cardiovascular disease, type 2 diabetes and cancer^2,3^. The benefits of physical activity and sleep also extend to mental health and enhanced cognitive functioning, such as improved learning, memory, and mental clarity^4–9^. Regular physical activity has been associated with greater emotional wellbeing, and even just one bout of physical activity can elevate mood^10^. On the other hand, poor sleep is associated with mood disorders and affective dysregulation^11^, and sleep disturbances are often comorbid with anxiety and depression^12,13^.

While the health benefits of physical activity and sleep are evident, the precise mechanisms and pathways by which these effects occur remain unclear. There is strong evidence that physical activity influences sleep^14^, suggesting that physical activity and sleep may act synergistically on health and psychological wellbeing. Therefore, a central goal is to understand how physical activity alters specific aspects of sleep and the cognitive and affective consequences of these changes. The existing literature suggests that both a single exercise session as well as sustained physical activity over time lead to changes in sleep quantity and quality^15^. Current evidence also suggests that both moderate-to-vigorous physical activity (e.g., running, fast cycling, fast swimming) as well as low-intensity physical activity (e.g., walking slowly, movement exercises or household chores) are associated with improvements in sleep quality, while increased sedentary behavior is associated with negative effects on sleep quality^14^. Moreover, sleep and physical activity have been theorized to have a bidirectional relationship over the short- and long-term^15^. Accordingly, good sleep could support consistent physical activity and more physical activity could improve sleep quality, potentially compounding their health benefits.

Lab studies have found exercise to significantly influence several sleep measures, including total sleep time (TST), decreased rapid-eye-movement (REM) sleep, higher sleep efficiency (SE), decreased wake-after-sleep-onset (WASO) and higher self-reported sleep quality (SSQ)^14,16^. However, changes in these metrics are seen with varying consistency and general conclusions are limited by differences in methodologies across studies^17^. Overall, the reduction in REM sleep has been one of the more consistent as well as unexpected findings^14,18–20^. Along with this decrease in total REM sleep, an increase in REM onset latency (REM-L) has often been observed following exercise, particularly when the session is proximal to sleep^21^. However, the mechanism of these effects on REM sleep and the behavioral and psychological consequences of these changes to sleep architecture are currently unknown.

To date, the effects of physical activity on sleep architecture have been studied almost exclusively in laboratory settings using polysomnography (PSG). PSG remains the gold-standard for sleep assessment, as it allows for the most accurate characterization of sleep stages using electroencephalography (EEG). However, this approach has several important limitations. First, due to high cost and personnel burden, PSG studies often measure an individual’s sleep on only one or a few nights, limiting the ability to measure sleep and variation in sleep over longer periods of time. Second, for the same reason, such studies are often also restricted to relatively small sample sizes. Third, and perhaps most importantly, laboratory studies require participants to sleep in an unfamiliar and potentially stressful clinical setting and are therefore unable to measure sleep as it occurs in natural sleeping environments. It is therefore important to extend laboratory-based research findings to larger sample sizes observed over longer time periods in naturalistic settings.

Recent advancements in the ability to passively monitor sleep and physical activity through wearable devices have opened the door to accomplishing this goal^22,23^. A first approach used actigraphy to assess the effects of physical activity on sleep, but this method is limited since actigraphy cannot distinguish different sleep stages. More recently, photoplethysmography (PPG) using optical heart rate monitors have made it possible to capture detailed measures of both ongoing physical activity as well as sleep, including detailed sleep architecture and specific sleep stages, outside of the lab^23^. A systematic review found that PPG-equipped devices had higher sensitivity (0.95–0.96) and specificity (0.58–0.69) for sleep epoch detection compared to actigraphy-only models^24^. Moreover, pooled estimates of several primary sleep metrics, including TST, SE and WASO, did not show statistically significant differences from PSG. While these are promising results, it is important to note that the accuracy of such devices for estimating sleep parameters varies across studies, may be less accurate than PSG for some metrics, have varied sensitivity and specificity for classifying specific sleep stages, and that more validation studies are needed (see section "Methods": Sleep for additional data about the accuracy of wearables in relation to PSG).

In the current study, we utilized wearable devices equipped with PPG coupled with smartphone ambulatory assessment to collect repeated daily measurements of sleep, physical activity, and mood in naturalistic settings. Data was obtained from two partially overlapping groups of participants (total N = 82) over two multi-month data collection intervals in one year, spanning a total data collection period of six months. The primary aims of the current study are to (1) evaluate the effects of physical activity on sleep architecture over time in naturalistic settings, and (2) characterize the psychological effects of physical activity and sleep architecture on mood and wellbeing in everyday life. We hypothesized that increased physical activity would be associated with changes in naturalistic sleep architecture similar to those observed in the lab, including longer TST, increased SE, decreased REM/NREM ratio and higher SSQ, and that these changes would in turn be associated with reduced stress and increased positive mood.

## Results

After data thresholding (see section "Methods"), the final sample for analysis consisted of 2287 days of observation from 65 participants (*M*_age_ = 21.40, *SD* = 3.21, N = 42 females) with an average of 35.20 (*SD* = 27.01) days of observation per participant. Each day of observation included estimates of physical activity for that day and sleep parameters/architecture for the corresponding night, derived from ambulatory assessment using wearable devices. In addition, self-reported mood during the evening preceding sleep and the morning after waking up were collected with ecological momentary assessments (EMAs) delivered through participants’ mobile devices. Descriptive statistics for person-level sleep, physical activity and mood metrics are summarized in Table 1. Altogether, participants submitted a total of 1259 evening EMAs and 1263 morning EMAs. On average, participants in the final sample wore the device for 23 h each day.

**Table 1:** Participant data characteristics.

	Median ± SD	Min	Max
Participant parameters			
Age, yr	20 ± 3.2	18	32
Device wear time, hr	23 ± 1.1	18	24
Physical activity parameters			
MVPA, min	16 ± 9.6	1	41
LPA, min	30 ± 12.9	5	72
SED, min	705 ± 80.3	531	912
Sleep parameters			
TST, min	400 ± 37.8	284	470
SE, %	93 ± 7.2	53	97
NREM sleep, min	310 ± 33.5	223	380
REM sleep, min	88 ± 16.6	41	127
REM / NREM ratio, %	28 ± 5.8	11	40
REM latency, min	105 ± 26.9	53	178
WASO, min	58 ± 10.8	26	83
Bedtime, time ± min	12:44 AM ± 109.5	5:16 PM	7:50 AM
Waketime, time ± min	8:26 AM ± 108.5	12:42 AM	4:31 PM
Morning mood parameters			
Content	1.6 ± 0.6	0	2.8
Stress	1.0 ± 0.6	0	3.0
Lonely	0.2 ± 0.5	0	1.8
Sad	0.3 ± 0.4	0	1.7
Energy	1.9 ± 0.5	0.7	3.2
Evening mood parameters			
Content	1.8 ± 0.6	0	3.0
Stress	1.0 ± 0.6	0	2.7
Lonely	0.3 ± 0.6	0	2.5
Sad	0.4 ± 0.4	0	1.5
Energy	1.9 ± 0.7	0	3.5

Parameters summarize person-level metrics averaged across all days in the study.

SED: Sedentary behavior (min), LPA: low-intensity physical activity, MVPA: moderate-to-vigorous physical activity. TST: total sleep time, SE: sleep efficiency, REM: rapid eye movement sleep, NREM: non-rapid eye movement sleep, WASO: wake after sleep onset.

The average duration of daily sedentary behavior, low-intensity physical activity (LPA), and moderate-to-vigorous-intensity physical activities (MVPA) were 702.5 min per day, 31.2 min per day, and 16.5 min per day, respectively. On average, participants spent 7.6 h in bed and had 6.6 h of total sleep time (TST) each night. 55% of all participants’ TSTs fell within the recommended range of 7–9 h per night. Sleep efficiency (SE) was generally high across the sample, with 96% of nights showing a SE greater than 80%. REM sleep accounted for an average of 21% of TST across the sample. Welch’s *t*-test results showed that males and females did not significantly differ on any mean daily measure of physical activity, sleep or mood (all *P* > 0.05).

Participants self-reported sleeping an average of 6.9 h per night, which is consistent with the device-measured 6.6 average hours of TST. Self-reported hours slept was highly correlated with device-measured TST (*r* = 0.74, *P* < 0.001). The median bedtime was 12:44 AM and the median wakeup time was 8:26 AM. 85% of participants reported an average sleep onset latency (SOL) less than 20 min, indicating optimal sleep initiation. 72% of participants reported the optimal amount of 1 or fewer number of awakenings (NAW) lasting longer than 5 min during the night. The median self-reported sleep quality (SSQ) was “somewhat restful” (score of 2 on 0–3 Likert scale). As expected, SSQ was positively associated with TST and SE and negatively associated with SOL and NAW (all *P* < 0.05).

### Physical activity and global sleep metrics

**Figure 1:**
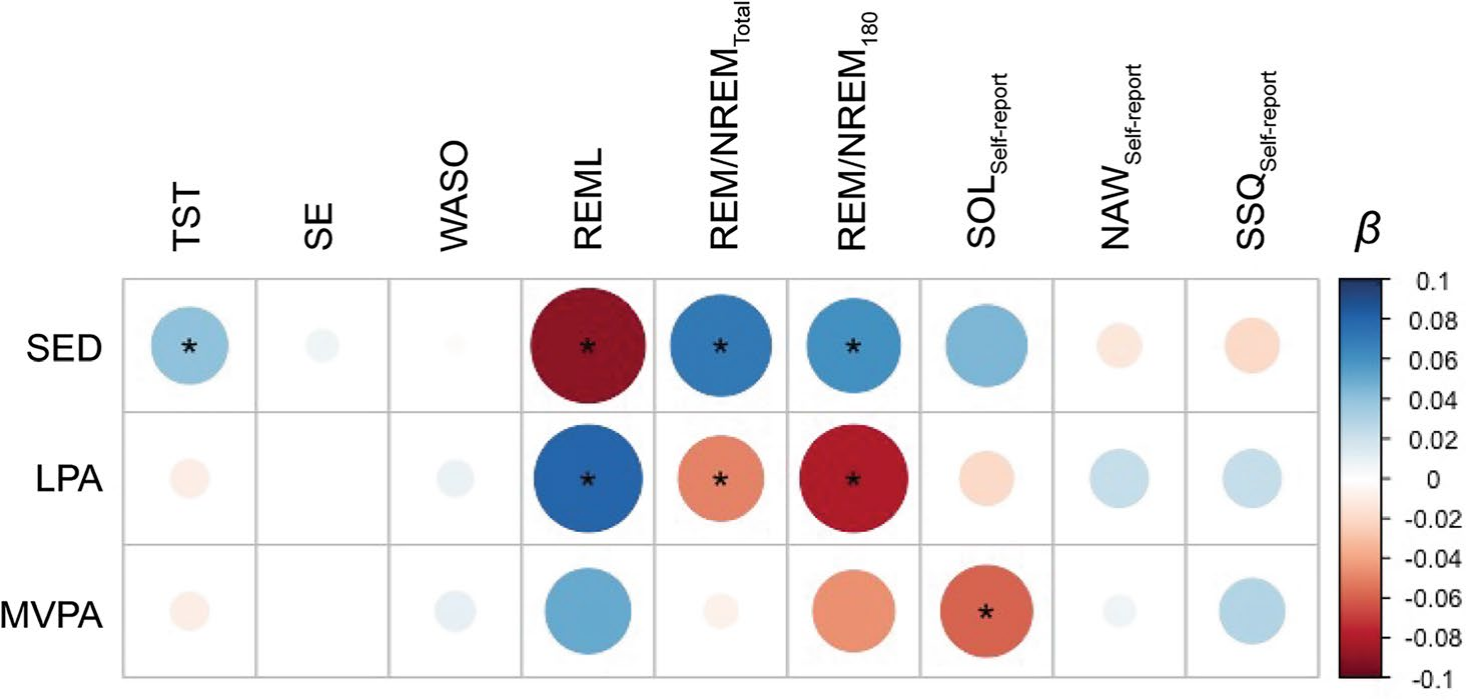
Within-person associations between previous day physical activity and overnight sleep. *Beta* = Standardized beta coefficients from linear mixed models. * Denotes significant after correcting for multiple comparisons (*p* < 0.05, FDR corrected). SED = Sedentary behavior (min), LPA = low-intensity physical activity, MVPA = moderate-to-vigorous physical activity. TST = total sleep time, SE = sleep efficiency, WASO = wake after sleep onset, REML = REM onset latency, REM / NREM_Total_ = ratio of REM to NREM sleep over the entire sleep interval, REM / NREM_180_ = ratio of REM to NREM sleep in the first 180 min following sleep onset, SOL = self-reported sleep onset latency, NAW = self-reported number of awakenings, SSQ = self-reported subjective sleep quality.

At the within-person level, TST was positively associated with the amount of previous-day sedentary (SED) behavior (*ß* = 0.05, *P* = 0.01), but not LPA (*ß* = − 0.01, *P* = 0.46) or MVPA (*ß* = − 0.01, *P* = 0.51). However, at the between-person level, TST was negatively associated with SED (*ß* = − 0.45, *P* < 0.001). MVPA (*ß* = − 0.06, *P* = 0.01) but not LPA (*ß* = − 0.02, *P* = 0.36) was associated with a shorter overall self-reported SOL, while SED was associated with a longer self-reported SOL (*ß* = 0.05, *P* = 0.03). No significant associations between physical activity and SE, WASO or self-reported NAW were observed at either the within-person or between-person level (all *P* > 0.05). SSQ was not associated with any physical activity metric at the within-person level (all *P* > 0.05) but was positively associated with MVPA at the between-person level (*ß* = 0.29, *P* = 0.02). Figure 1 summarizes the within-person associations between previous-day physical activity and overnight sleep metrics. No overnight sleep metric was significantly associated with the amount of LPA or MVPA on the next day (the day following the sleep episode) (all *P* > 0.05); thus, we did not find evidence for bidirectional effects of sleep and physical activity.

We next evaluated the influence of engaging in at least 60 min of MVPA during the day on overnight sleep, mirroring a physical activity condition in an experimental sleep laboratory study (see section "Methods"). Engaging in at least 60 min of MVPA was associated with higher SE (*ß* = 0.03, *P* = 0.03), shorter SOL (*ß* = − 0.06, *P* = 0.006), higher SSQ (*ß* = 0.09, *P* = 0.0009), and a trend toward reduced WASO (*ß* = − 0.02, *P* = 0.1). In summary, with the exception of self-reported SOL, continuous measures of LPA and MVPA were not significantly associated with most global metrics of sleep quality, but engaging in at least 60 min of MVPA was associated with improvements in several markers of global subjective and objective sleep quality.

### Physical activity and sleep architecture

Over the total sleep period, previous-day SED was associated with a decreased percentage of NREM sleep (*ß* = − 0.06, *P* = 0.002), an increased percentage of REM sleep (*ß* = 0.06, *P* = 0.002) and an increased REM/NREM ratio (*ß* = 0.07, *P* = 0.001; Fig. 1). LPA showed the opposite pattern and was associated with an increased NREM percentage (*ß* = 0.06, *P* = 0.004), a decreased REM percentage (*ß* = − 0.06, *P* = 0.004) and a lower REM/NREM ratio (*ß* = − 0.06, *P* = 0.004; Fig. 1). MVPA did not show a significant association with REM or NREM percentage over the entire sleep interval (all *P* > 0.05).

A similar pattern was observed for the first 180 min following sleep onset: SED was again associated with a higher REM/NREM ratio (*ß* = 0.06, *P* = 0.01), while both LPA (*ß* = − 0.08, *P* = 0.005) and MVPA (*ß* = − 0.05, *P* = 0.05) were associated with a lower REM/NREM ratio. Furthermore, SED was associated with a shorter REM-L (*ß* = − 0.09, *P* < 0.001), while LPA (*ß* = 0.08, *P* = 0.003) and MVPA (*ß* = 0.05, *P* = 0.02) were both associated with a longer REM-L. Follow-up analysis revealed that time-of-day of physical activity was a significant moderator: longer REM-L was specifically associated with evening LPA (*ß* = 0.07, *P* = 0.01) and MVPA (*ß* = 0.06, *P* = 0.01). Correspondingly, time-of-day moderated the influence of LPA and MVPA on decreased REM/NREM ratio in the first 180 min of sleep (evening LPA: *ß* = -0.09, *P* < 0.001, and MVPA: *ß* = -0.07, *P* = 0.007).

Neither LPA (*ß* = − 0.001, *P* = 0.96) nor MVPA (*ß* = 0.02, *P* = 0.42) showed a significant association with sleep onset time. However, higher amounts of LPA (*ß* = 0.05, *P* = 0.03) and MVPA (*ß* = 0.05, *P* = 0.03) were associated with an earlier wake-up time the previous morning. Later sleep onset time was associated with reduced TST (*ß* = − 0.49, *P* < 0.001) as well as a higher REM/NREM ratio in the first 180 min of sleep (*ß* = 0.09, *P* = 0.002) and a shorter REM-L (*ß* = − 0.09, *P* = 0.002). Earlier wake-up time the previous morning was also associated with a higher REM/NREM ratio (*ß* = 0.10, *P* < 0.001) as well as a shorter REM-L (*ß* = − 0.07, *P* = 0.02), but was not associated with TST (*ß* = 0.03, *P* = 0.37). To account for the influence of sleep timing on sleep architecture, we performed a follow-up analysis including sleep onset time and previous-day wake-up time as covariates in the overall model. The same pattern of results was observed with sleep timing covariates included. As before, LPA was associated with a lower REM/NREM ratio (Overall: *ß* = −0.08, *P* < 0.001; First 180 min: *ß* = − 0.08, *P* = 0.01), while MVPA was again only significantly associated with a lower REM/NREM ratio in the first 180 min of sleep (Overall: *ß* = − 0.03, *P* = 0.14; First 180 min: *ß* = − 0.07, *P* = 0.02). SED was again associated with a higher REM/NREM ratio, but in this model only reached significance for the entire sleep period (Overall: *ß* = 0.09, *P* = 0.002; First 180 min: *ß* = 0.04, *P* = 0.21). Finally, SED was again associated with a shorter REM-L (*ß* = − 0.09, *P* = 0.007), while LPA (*ß* = 0.07, *P* = 0.01) and MVPA (*ß* = 0.06, *P* = 0.04) were both associated with a longer REM-L.

The overall pattern of REM-L and physical activity was also observed at the between-person level: higher average SED time was associated with shorter average REM-L (*ß* = − 0.37, *P* = 0.02), while higher average LPA (*ß* = 0.32, *P* = 0.04) and MVPA (*ß* = 0.35, *P* = 0.03) were associated with longer average REM-L. At the between-person level, the REM/NREM ratio during the first 180 min was significantly associated with average SED (*ß* = 0.32, *P* = 0.04), but not with average MVPA or LPA (*P* > 0.05). The average REM/NREM ratio over the entire sleep interval was not associated with average levels of physical activity at the between-person level (all *P* > 0.05). The REM/NREM ratio in the first 180 min was also negatively associated with SSQ (*ß* = − 0.44, *P* = 0.005), but not the REM/NREM ratio over the entire sleep period (*P* > 0.05).

In summary, sedentary behavior was associated with a higher REM/NREM ratio and a shorter REM onset latency, while LPA and MVPA were associated with a longer REM onset latency and a lower REM/NREM ratio, particularly in the first 3 h of sleep.

### Sleep and mood

**Figure 2:**
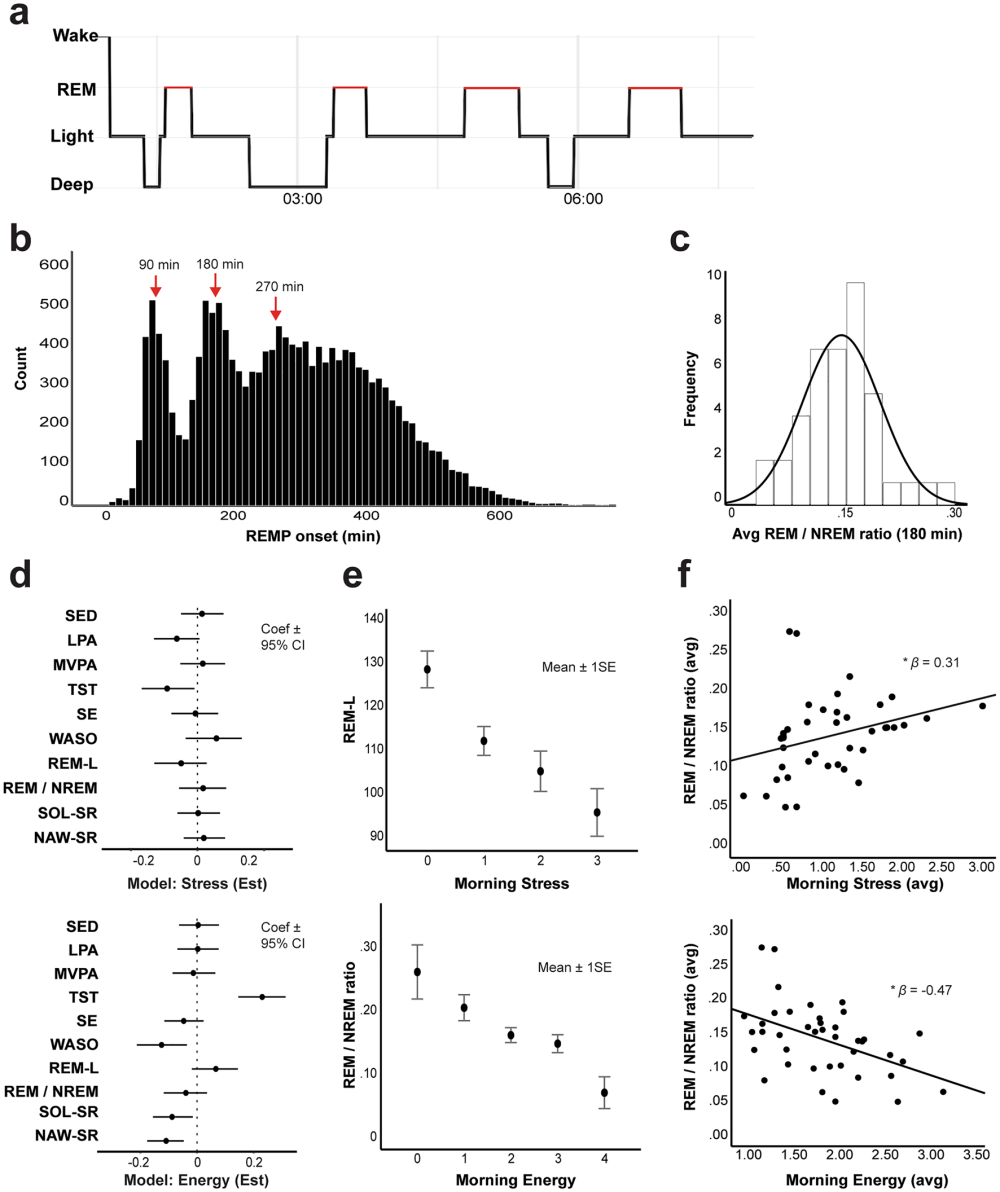
Sleep architecture characteristics and relationship to mood. (**a**) Hypnogram from a representative participant. Black lines indicate sleep stages; vertical gray lines indicate 90-min increments; red horizontal lines indicate REM periods. (**b**) Histogram of all REM period (REMP) onset times for all participants. Characteristic peaks are observed at 90, 180 and 270 min for the onset of the first three REM periods, corresponding to ultradian cycles. (**c**) Histogram of the average REM/NREM ratio in the first 180 min of sleep for all participants. (**d**) Simultaneous multiple regression models predicting morning stress (top) and energy (bottom) from sleep and physical activity. Dots indicate estimated coefficients and error bars indicate 95% confidence intervals. (**e**) Standard error plots showing inverse relationship between REM-L and morning stress (top) and REM/NREM ratio and morning energy (bottom). Dots represent average values of REM-L and REM/NREM ratios, respectively, and error bars represent standard errors of the mean. (**f**) Between-person correlations between the average REM/NREM ratio and average morning stress (top) and average morning energy (bottom).

Figure 2 summarizes the distribution of device-measured sleep architecture characteristics and their relationship to mood. Within-person associations between overnight sleep and morning mood are summarized in Table 2. Positive affective states (contentment, energy) were both associated with longer TST, shorter self-reported SOL, fewer self-reported NAW, and higher SSQ (all *P* < 0.05; Table 2). In contrast, negative affective states (loneliness, sadness) were associated with a longer self-reported SOL, greater self-reported NAW, and lower SSQ (all *P* < 0.05; Table 2). Lower morning stress was associated with longer TST and higher SSQ (*P* < 0.01). At the between-person level, higher average SSQ was associated with higher average contentment and energy and lower average loneliness, sadness and stress (all *P* < 0.05).

**Table 2:** Within-person associations between overnight sleep and morning mood.

	Content		Stress		Lonely		Sad		Energy	
	*Beta*	*p*	*Beta*	*p*	*Beta*	*p*	*Beta*	*p*	*Beta*	*p*
TST	0.12	< 0.001*	− 0.06	0.004*	− 0.006	0.77	− 0.017	0.48	0.156	< 0.001*
SE	0.03	0.16	− 0.001	0.98	− 0.03	0.19	− 0.067	0.01*	0.035	0.17
WASO	− 0.01	0.66	0.01	0.52	0.03	0.21	0.05	0.04	− 0.008	0.74
REM-L	0.05	0.1	− 0.08	0.01*	− 0.03	0.37	− 0.026	0.39	0.075	0.03
REM/NREM_TOT_	0.03	0.31	0.04	0.21	0.03	0.32	− 0.025	0.45	0.063	0.06
REM/NREM_180_	− 0.02	0.41	0.05	0.13	0.05	0.08	− 0.001	0.97	− 0.076	0.01*
SOL_Self-report_	− 0.07	0.008*	0.04	0.15	0.05	0.03	0.09	< 0.001*	− 0.058	0.03
NAW_Self-report_	− 0.09	0.002*	0.02	0.32	0.08	< 0.001*	0.059	0.02	− 0.099	< 0.001*
SSQ_Self-report_	0.29	< 0.001*	− 0.15	< 0.001*	− 0.07	0.003*	− 0.155	< 0.001*	0.36	< 0.001*

Beta: Standardized beta coefficients from linear mixed models. P: uncorrected two-tailed *p*-value. * Denotes significant after correcting for multiple comparisons (*p* < 0.05, FDR corrected). SED: Sedentary behavior (min), LPA: low-intensity physical activity, MVPA: moderate-to-vigorous physical activity. TST: total sleep time, SE: sleep efficiency, WASO: wake after sleep onset, REM-L: REM onset latency, REM/NREM_Tot_: ratio of REM to NREM sleep over the entire sleep interval, REM/NREM_180 min_: ratio of REM to NREM sleep in the first 180 min following sleep onset, SOL_Self-report_: self-reported sleep onset latency, NAW_Self-report_: self-reported number of awakenings, SSQ_Self-report_: self-reported subjective sleep quality.

At the within-person level, there was also a significant association between longer REM-L and reduced morning stress (*ß* = − 0.08, *P* = 0.01; Fig. 2e, top) as well as increased energy (*ß* = 0.08, *P* = 0.03). Conversely, a higher REM/NREM ratio in the first 180 min of sleep was associated with lower morning energy (*ß* = − 0.08, *P* = 0.01; Fig. 2e, bottom). People with longer average REM-L also had lower average morning stress (*ß* = − 0.35, *P* = 0.03) and marginally higher average energy (*ß* = − 0.31, *P* = 0.056). Additionally, people with a higher average REM/NREM ratio in the first 180 min of sleep had higher average morning stress (*ß* = 0.31, *P* = 0.04; Fig. 2f, top), and lower average energy (*ß* = − 0.47, *P* = 0.002; Fig. 2f, bottom) and contentment (*ß* = − 0.34, *P* = 0.03). Total number of awakenings was associated with higher average morning stress (*ß* = 0.27, *P* = 0.03), average increased loneliness (*ß* = 0.25, *P* = 0.04) and lower average energy (*ß* = − 0.25, *P* = 0.04). In contrast to the findings for morning mood, no measures of evening mood preceding the sleep episode were significantly associated with any sleep architecture variable after correcting for multiple comparisons (all *P* > 0.05).

In summary: (1) TST was associated with positive affect (increased contentment and energy) and reduced stress; (2) Sleep architecture changes associated with physical activity (i.e., longer REM-L and lower REM/NREM ratio) were associated with positive affective states and reduced stress at both the within and between-person level; (3) Sleep markers associated with difficulty falling and remaining asleep, such as self-reported NAW and SOL, were associated with negative affective states such as loneliness and sadness.

### Physical activity and mood

Within-person associations between physical activity and evening and morning mood are summarized in Table 3. Within-person levels of sedentary behavior were associated with numerically higher stress and lower energy in the evening; however, neither of these effects remained significant after multiple comparisons corrections.

**Table 3:** Within-person associations between physical activity and evening and morning mood.

Evening	Content		Stress		Lonely		Sad		Energy	
	*Beta*	*p*	*Beta*	*p*	*Beta*	*p*	*Beta*	*p*	*Beta*	*p*
SED	− 0.02	0.50	0.06	0.01	0.02	0.35	0.03	0.18	− 0.06	0.03
LPA	0.03	0.21	− 0.02	0.30	− 0.03	0.13	− 0.01	0.77	0.03	0.27
MVPA	0.03	0.14	− 0.017	0.47	− 0.04	0.09	− 0.003	0.87	0.04	0.11

Morning	Content		Stress		Lonely		Sad		Energy	
	*Beta*	*p*	*Beta*	*p*	*Beta*	*p*	*Beta*	*p*	*Beta*	*p*
SED	− 0.03	0.26	0.05	0.03	0.06	0.01	0.04	0.08	0.02	0.48
LPA	0.01	0.54	− 0.08	0.0009*	− 0.05	0.03	− 0.03	0.19	0.003	0.90
MVPA	− 0.01	0.74	− 0.03	0.17	− 0.009	0.70	− 0.01	0.70	− 0.001	0.99

Beta: Standardized beta coefficients from linear mixed models. p: uncorrected two-tailed *p*-value. * Denotes significant after correcting for multiple comparisons (*p* < 0.05, FDR corrected). SED: Sedentary behavior (min), LPA: low-intensity physical activity, MVPA: moderate-to-vigorous physical activity.

Lower morning stress was associated with higher amounts of LPA the previous day (*ß* = − 0.08, *P* < 0.001) and higher stress was associated with more previous-day SED behavior (*ß* = 0.05, *P* = 0.03), but stress was not significantly associated with previous-day MVPA (*ß* = − 0.03, *P* = 0.17). The effects of LPA on morning stress remained significant when including TST, REM-L and SSQ as covariates (*P* < 0.05; Fig. 2D). Higher levels of loneliness were also positively associated with SED (*ß* = 0.06, *P* = 0.01) and negatively associated with LPA (*ß* = − 0.05, *P* = 0.03; Table 3), but these effects were not significant after correcting for multiple comparisons. At the between-person level, higher average levels of both LPA and MVPA were associated with higher average levels of both contentment (LPA: *ß* = 0.26, *P* = 0.03; MVPA: *ß* = 0.43, *P* < 0.001) and energy (LPA: *ß* = 0.29, *P* = 0.02; MVPA: *ß* = 0.39, *P* < 0.001).

As LPA was significantly associated with both REM-L and the percentage of NREM sleep in the first 180 min, and each of these measures was in turn significantly associated with reduced morning stress, we evaluated whether REM-L and NREM% mediated the relationship between previous day physical activity and morning stress. However, we did not find evidence for mediation: in both models LPA remained as a significant predictor of morning stress when including REM-L or NREM% as a simultaneous predictor (all *P* < 0.05). Figure 2d shows estimates and 95% confidence intervals for physical activity and sleep variables as simultaneous predictors of morning stress (top) and energy (bottom).

In summary, at the within-person level, psychological stress was the only dimension significantly associated with physical activity, which was lower for higher amounts of previous-day LPA. Improvements in sleep did not mediate the relationship between LPA and stress, indicating that physical activity and sleep account for unique variance in morning stress.

## Discussion

The primary aim of this study was to evaluate the effects of physical activity on sleep architecture in naturalistic environments and the associated effects on psychological mood. We observed a robust inverse relationship between sedentary behavior and physical activity on sleep architecture. Sedentary behavior was associated with decreased NREM sleep, increased REM sleep, and a shorter REM sleep onset latency. Conversely, both light and moderate-to-vigorous physical activity showed the opposite pattern: increased NREM sleep, decreased REM sleep and a longer REM latency (REM-L). These changes in REM and NREM sleep were also observed during the first 180 min of sleep, indicating that they were not attributable to alterations in total sleep time, and were also robust to differences in sleep timing. These findings are consistent with laboratory PSG studies that have evaluated the effects of exercise on REM/NREM sleep architecture, which have found that exercise reduces REM sleep and increases REM-L^14,16,21,25,26^. Our results extend these findings to naturalistic environments and demonstrate that these changes in sleep architecture can be observed over time within individuals. Notably, while lab-based studies have predominantly demonstrated one-day prior effects, the current results show that these relationships remain consistent over a period of at least multiple weeks to months.

The psychological consequences of these alterations in sleep architecture have not previously been evaluated and the implications for sleep quality have also remained unclear. The current analysis suggests that these changes are associated with positive effects on both mood and sleep quality. Specifically, we observed that longer REM-L was associated with decreased stress and increased energy the morning after sleep. Furthermore, a higher REM/NREM ratio in the first 180 min of sleep was associated with lower morning energy and worse self-reported sleep quality (SSQ). Thus, the decrease in the REM/NREM ratio that is associated with physical activity, particularly in the first 3 h after sleep onset, is associated with more energy in the morning after sleep as well as increased perceived restfulness of sleep. Intriguingly, these findings parallel PSG studies of depressed patients which show the opposite pattern of a disinhibition of REM sleep, including a shorter REM latency, prolonged REM sleep early in the night, and increased REM density^27^. As reductions in REM sleep and increased REM-L are observed as a consequence of antidepressant medications^28^, the changes in REM sleep as a result of physical activity warrant further attention as a potential antidepressant mechanism. While no significant associations between REM-L or the whole-night REM/NREM ratio and depressed affect were observed in the current study, a higher REM/NREM ratio in the first 180 min was marginally associated with increased loneliness. It would be useful in future work to test how these sleep architecture variables correlate with depression subtypes and symptoms in clinical populations or using validated questionnaires.

In addition to the effects of physical activity on sleep architecture, we also observed several associations between daily physical activity and global sleep indices. Consistent with previous studies, we observed that moderate-to-vigorous physical activity was associated with a shorter sleep onset latency while sedentary behavior was associated with a longer sleep onset latency. Regression models that included continuous physical activity measures yielded relatively weak results with other global metrics of sleep, such as sleep efficiency and wake-after-sleep-onset, which have been found to be influenced by exercise in laboratory sleep studies. However, our data show that engaging in at least 60 min of moderate-to-vigorous physical activity was associated with improvements in several global sleep metrics, including increased sleep efficiency, shorter sleep onset latency, higher subjective sleep quality (SSQ)/restfulness of sleep, as well as a trend toward decreased wake-after-sleep-onset. Given that this quantity of physical activity is comparable with a physical activity condition in experimental studies, these results may be taken to be consistent with findings of previous laboratory studies. Together, the results support that engaging in a substantial amount of moderate-to-vigorous physical activity is associated with global improvements in sleep, including higher sleep efficiency and subjective sleep quality.

These findings provide a crucial extension of effects observed in the laboratory to naturalistic sleep environments. The results regarding sleep onset latency and sleep efficiency are particularly noteworthy given that both of these metrics are associated with increased mortality^29^. The current results fill a much needed gap in the literature by showing that these effects remain valid in people’s natural sleeping environments and are observed as a consequence of the physical activity that people engage in in their everyday lives. The fact that high levels of moderate-to-vigorous physical activity increases perceived sleep quality is also of note given that SSQ has been found to predict increased life satisfaction and improved mental health^30^. Indeed, the current data provide further support for the link between perceived sleep quality and psychological wellbeing: positive affective states of energy and contentment were positively correlated with SSQ, while negative affective states of sadness, loneliness and stress were negatively correlated with the SSQ at both the within and between-person level.

In contrast to several previous studies, we did not observe an association between either light or moderate-to-vigorous physical activity and increased total sleep time^14^. Furthermore, while sedentary behavior was associated with less total sleep time at the between-person level, sedentary behavior was positively associated with total sleep time at the within-person level. These results imply that people who tend to be sedentary on average also tend, on average, to get less sleep, while, in contrast, for a given person, sleeping relatively longer on a given day is also associated with a relatively high amount of sedentary activity the previous day. While this finding was unexpected, and this analysis was not the primary aim of the current study, we note that it is possible for between-person and within-person effects to show opposing relationships, and such opposing effects are not uncommon^31^. One possibility is that the between-person effect reflects lifestyle factors such as general tendencies toward healthy behaviors (i.e., those who tend to prioritize being physically active also prioritize getting sufficient sleep), whereas the within-person effect reflects a different process related to the physiological effects of sedentary behavior on sleep described above. Notably, screen-time sedentary behaviors have been associated with a longer sleep duration^32^, suggesting that certain types of sedentary behaviors may be predictive of TST while others are not. Thus, an alternative possibility is that different types of sedentary behavior drive the opposing within and between-subjects effects. While a precise explanation of these effects requires further research, these opposing effects may help explain why the link between TST and physical activity has been observed in some studies but not others^19,33^.

The only psychological dimension that was significantly associated with physical activity in the current study was morning psychological stress. Specifically, we observed that higher amounts of low-intensity physical activity were associated with lower levels of stress the next morning. Low stress levels were also linked to several sleep metrics, including increased TST, increased percentage of NREM sleep in the first 3 h of sleep, a longer REM-L and a higher subjective sleep quality. However, we did not find evidence that any of these sleep metrics mediated the relationship between previous-day physical activity and stress. Our results therefore suggest that increased physical activity and improved sleep quality are each associated with reductions in psychological stress and that each account for unique variance in stress reduction. The finding that lower stress is linked to improved sleep quality and increased physical activity is consistent with several previous studies that have evaluated the link between physical activity, sleep and stress. For instance, a longitudinal study found that higher sleep quality and longer sleep duration the night before an exam significantly predicted less stress experience during the exam^34^. Furthermore, a behavioral health intervention targeting sleep and physical activity among physically inactive and poor sleeping adults found that when these behaviors improved, so did their psychological stress^35^. Given that low-intensity physical activity more frequently involves social activities and relationships^36^, it will be important in future work to evaluate to what extent the social component of these activities contributes to stress reduction.

The mechanism of the effects of physical activity on REM and NREM sleep architecture remains unclear. Previous lab studies have found that both low-intensity and high-intensity physical activity decreases REM sleep and increase REM latency^21^. Moreover, consistent with the present results, the effects of physical activity on REM sleep percentage and latency in prior studies have been most pronounced when the activity occurs in the evening hours more proximal to bedtime^14,16,21^. One possible explanation for these effects invokes the concept of restorative sleep, in which deep NREM sleep provides a recovery period from metabolic stress^37^. In this view, physical activity promotes increased NREM sleep, which has the indirect consequence of delaying REM sleep until later in the night. A related hypothesis is that increased core body temperature due to physical activity increases the propensity for NREM sleep and suppresses REM sleep^38^, which would be consistent with the larger influence of evening exercise. Alternatively, physical activity could directly inhibit or delay REM sleep. One possibility for how this could occur is through increased aminergic neurotransmitter levels and corresponding sympathetic activity^39^. In support of this, a study of REM sleep in athletes found that increases in REM latency as a result of exercise were correlated with norepinephrine excretion rates^26^. Finally, given that the timing of REM sleep is governed by the circadian system^40^, it is possible that prolonged REM latency is caused by circadian rhythm phase delays^16^. The above hypotheses are not mutually exclusive, and it is possible that there could be multiple pathways by which these effects occur. Overall, more research is needed to determine the physiological mechanisms of the changes in REM and NREM sleep architecture associated with physical activity.

For the first time, wearable devices equipped with optical heart rate monitors, such as the devices used in the current study, have made it possible to measure detailed sleep architecture in natural environments, and have opened the door to critical new opportunities for research outside the traditional laboratory environment. A meta-analysis of validation studies of sleep assessment in Fitbit models suggests that recent-generation (post-2017) sleep-staging models, which all share the same core hardware and algorithms, found that many sleep parameters, including TST, SE and WASO, have clinically negligible estimation bias compared to PSG^24^. For instance, device-measured and PSG-measured TST differed by less than 12 min across studies. However, the accuracy of sleep stage parameters was more variable: 0.69–0.81 for light sleep, 0.36–0.89 for deep sleep, and 0.62–0.89 for REM sleep. The existing studies taken together therefore suggest that these devices are imperfect in sleep classification and are not a substitute for gold-standard PSG^41^. Nevertheless, the accuracy of recent-generation wearables that combine actigraphy with optical plethysmography are a substantial improvement over earlier models and show promise for use in many applied settings. Finally, it is important to note that there are currently few validation studies for recent-generation models and additional validation data is needed on the accuracy and reliability of wearable devices for sleep staging in different settings and populations.

A limitation of the current study is that we did not distinguish between different categories or types of physical activity. It is plausible that some types of activities may preferentially improve sleep or mood, and identifying these activities would be useful in future research. For instance, as noted above, the finding that low-intensity physical activity appears preferentially linked to stress reduction raises the question of whether activities with a social component may have additional benefits for mood, mental health and/or sleep. Furthermore, as occupational and leisure physical activity can differentially influence mood^19^, distinguishing between these categories of activity will be useful in future research. It is also important to note that the current sample was restricted to young adults. It will be important in future research to collect data from more diverse samples and to evaluate how the effects could vary with age and other socioeconomic factors. Finally, while the current results suggest that the effects of physical activity on sleep architecture persist over time, future research conducted over multiple years, such as longitudinal cohort studies, would be valuable to study these effects over even longer time intervals.

Together, the current results show that physical activity exerts measurable changes in sleep architecture in naturalistic settings, including increasing the percentage of time in NREM sleep, decreasing the percentage of time in REM sleep and increasing REM sleep latency. These changes in sleep architecture are in turn associated with improved wellbeing, including increased energy, reduced stress and enhanced perceived restfulness of sleep. Physical activity, particularly low-intensity physical activity, as well as the quality and quantity of overnight sleep, appear to account for unique variance in psychological stress reduction. Understanding the neurobiological mechanisms of these changes in REM and NREM sleep architecture as well as their effects on behavior and cognition will be an exciting opportunity for future research.

## Methods

### Participants

Eighty two undergraduate students enrolled at The University of Texas at Austin (UT) between 18 and 35 years of age participated in the study. The study was approved by the UT Austin IRB (study number 2019-09-0120). The study was conducted in accordance with the Declaration of Helsinki and all relevant guidelines and regulations. All participants provided signed informed consent prior to the study. Prior to consenting to participate, all participants were screened for eligibility via a virtual enrollment interview. Exclusion criteria included current neurological or psychiatric or psychological disorders, significant substance abuse or hormone altering medication intake. Recruitment occurred over two distinct periods. The first group of 76 participants was recruited in early January 2020 before the spring semester started, and data collection ran from mid-January to late March when Phase 1 data collection was halted due to the onset of COVID-19. The second group consisted of 37 participants—31 of which also participated in Phase 1—enrolled during the two weeks prior to May 1st, 2020. Phase 2 data collection began in May and ended in early September 2020, after all participants had scheduled a virtual exit interview with the study coordinator and arranged to ship study materials back to UT. All Phase 1 participants’ primary residence was in Austin during the study, whereas some Phase 2 participants resided elsewhere in Texas during the study.

### Ecological momentary assessments (EMAs)

The Beiwe™ smartphone app and research platform^35^ was used to collect real-time experience-sampling data, also referred to as Ecological Momentary Assessments (EMAs). EMAs were collected twice per day, once in the morning and once in the evening. EMAs were distributed on Monday, Wednesday, Friday, and Sunday in the mornings at 9:00 am and evenings at 7:00 pm. EMAs asked participants to self-report about their previous night’s sleep in the morning and about their mood in both the morning and evening. Participants rated their current psychological state on 5 different dimensions of mood/wellbeing: contentment, stress, loneliness, sadness and energy. These dimensions were established through previous research using EMAs in multimodal remote data collection^42^. Contentment, stress, loneliness and sadness were rated on a 0–3 Likert scale from 0 (*not at all*) to 3 (*very much*). Energy was rated on a 0–4 Likert scale that ranged from low energy (0) to neutral (2) to high energy (4).

### Sleep

Sleep and physical activity data were collected using the Fitbit Inspire HR, a wrist-worn wearable device equipped with an accelerometer and optical heart rate monitor. Proprietary Fitbit algorithms convert accelerometer and heart rate data into sleep and physical activity measures. Participants’ Fitbit data was uploaded to the Fitabase server, from which we retrieved daily sleep and activity measures as well as minute-level heart rate data. Participants were encouraged to wear their Fitbit as often as possible, only removing it to charge.

The Fitbit Inspire HR is able to determine sleep stage transitions, and therefore total time in each sleep stage, using a combination of heart-rate and movement data^43^. Sleep metric variables included total sleep time (TST), wake after sleep onset (WASO), sleep efficiency (SE), minutes of light sleep, minutes of deep sleep, minutes of REM sleep, and REM onset latency (how long after initial sleep onset that REM sleep begins, REM-L). TST and other sleep variables were calculated from the main overnight sleep period and did not include daytime naps.

As noted above, a meta-analysis of validation studies of sleep assessment in Fitbit models suggests that recent-generation sleep-staging models have a negligible estimation bias for TST, SE and WASO compared to PSG^24^. However, it is important to note that epoch-by-epoch (EBE) accuracy of sleep stage classification compared to PSG has been conducted in only handful of studies, and these amalgamated results were only based on three published studies^44–46^. Moreover, individual studies differ in the observed discrepancies between device-measured and PSG-derived sleep metrics. For instance, two of the three studies reported that Fitbit devices significantly overestimated TST by approximately 10 min, while the third study found nonsignificant overestimation. Only one study compared SE and found that devices overestimated SE by approximately 2%. With respect to sleep stage classification, accuracy across studies varied from 0.69 and 0.81 for detecting light NREM sleep (stages 1 and 2), 0.36 and 0.89 for detecting deep NREM sleep (stage 3), and 0.62 and 0.89 in detecting REM sleep.

Since this systematic review, an additional validation study has been published on Fitbit Inspire 2™, a device highly similar to the one used in the present study. This study found that Inspire 2™ significantly overestimated TST, deep sleep and REM sleep compared to PSG. However, the discrepancies in total time were relatively small (18 min for TST, 15 min for deep sleep and 9 min for REM sleep) and the effects sizes were also small. SE and WASO were also numerically overestimated but these differences were not significant. EBE accuracy was 59.1% for light NREM sleep, 83.7% for deep NREM sleep and 82.3% for REM sleep. Collectively these studies indicate that wearable devices similar to those used in the present analysis have good overall sensitivity for sleep, but may slightly overestimate TST, and have decent but not perfect accuracy for REM sleep and NREM sleep classification. Inspection of the confusion matrices in the above studies show that low accuracy for light NREM or deep NREM is often attributable to inability of devices to accurately distinguish between light and deep NREM. To account for this limitation, in the current study we combined both variables into a NREM sleep variable which was computed as the sum of light NREM sleep and deep NREM sleep stages.

Self-report sleep measures for the previous night’s sleep were also obtained from morning EMAs. These included estimates of sleep duration, time in bed until initial sleep onset (sleep onset latency or SOL), the number of awakenings during the night (NAW), and self-perceived sleep quality/restfulness of sleep overall (SSQ). Participants recorded their sleep duration in 1-h increments, and we numerically represented the responses as the middle of the range (0: *0 h*, 1.5: *1–2 h*, 2.5: *2–3 h,* up until 12: *more than 12 h*). For SOL, participants responded on a scale from 0 to 3 (0: *less than 10 min*, 1: *10–20 min*, 2: *20–30 min*, 3: *more than 30 min*). Participants also recorded their NAW on a scale from 0 to 3 (0: *0–1 awakenings*, 1: *2 awakenings*, 2: *3–4 awakenings*, 3: *more than 4 awakenings*). Participants responded to the question, “How restful was your sleep last night?” on a scale from 0 (*not at all restful*) to 3 (*very restful*). During the second period of data collection, participants were able to respond to the sleep duration, SOL, and NAW questions with any number, but these numbers were converted to the same scale as above to facilitate combined analysis across both groups.

### Physical activity

Physical activity data was measured using Fitbit Inspire HR wristwatches. Fitbit uses proprietary algorithms to convert raw sensor data from wristwatches into physical activity measures, some of which we used directly in analyses. The Inspire HR calculates lightly active, fairly active, and very active minutes—collectively called Active Zone minutes. Active Zone Minutes are based on target heart rate zones and are designed in accordance with the *Physical Activity Guidelines for Americans, 2nd edition.* At the time of this study, most physical activity monitors used age-predicted maximal heart rate (APMHR) to determine target heart rate zones, including the model used in this study. However, some newer Fitbit models now use heart rate reserve (HRR) instead. HRR is the difference between a person’s maximal heart rate (MHR) and resting heart rate (RHR).

As there is evidence that HRR provides a more optimal method to calculate target heart rate zones^47^, we used this method in the current analysis. Specifically, we used HRR to calculate the duration of light and moderate-to-vigorous physical activity (LPA and MVPA, respectively) for the following reasons: (1) there are inconsistent target heart rate zones between various activity monitors that don’t align with cardiovascular intensity classifications, (2) to adopt the improved method used to calculate target heart rate zones by newer activity monitors, and (3) to measure a broader range of physical activity than is possible using step-based activity, which may exclude other forms of physical activity that elevate heart rate.

To calculate physical activity metrics using HRR, we retrieved heart rate data measured in approximately 10-s (second cohort) to 1-min (first cohort) intervals and daily RHR measures estimated by Fitbit. Then, we estimated each participant’s maximal heart rate (MHR) using the age-predicted maximal heart rate equation (APMHR = 220–age). Next, we calculated each participant’s HRR, using the formula HRR = APMHR–RHR. The formula to calculate target heart rate zones is: HRR * (% of desired intensity) + RHR. Lastly, we calculated the daily time within each target heart rate zone by summing the time within each zone (excluding in-range heart rate measurements with a difference between samples of greater than 60 s as this could indicate a gap in wear time). To evaluate the effects of physical activity during specific times of day, we also calculated the duration of LPA and MVPA separately in the morning (6 am to 12 pm), afternoon (12 to 6 pm), and evening (6 pm to 12 am) using the same process.

### Statistical analysis

In order to ensure data quality, daily observations had to meet the following criteria: (1) >  = 18 h of wear time to ensure that both the sleep period and waking activity were adequately sampled; (2) TST of >  = 3 h, as this is the minimum required time for Fitbit to calculate sleep stages; (3) sleep onset initiated between 5 pm and 8 am (to exclude daytime naps); (4) morning EMAs completed between 5 am and 3 pm and evening EMAs completed between 5 pm and 3 am (to ensure EMAs were accurately capturing in-the-moment reports of current states rather than retrospective reports).

To account for varied numbers of repeated observations within participants, we used a linear mixed effects model to evaluate repeated-measures effects of physical activity (PA) on sleep and mood and the effect of sleep on mood. The model used restricted maximum likelihood estimation (REML). Shapiro–Wilk tests indicated that LPA and MVPA measures significantly deviated from a normal distribution (LPA: Shapiro–Wilk’s *W* = 0.83, *P* < 0.001; MVPA: *W* = 0.58, *P* < 0.001). We therefore used nonparametric bootstrapping for significance tests of mixed-model regression coefficients. Hypothesis testing of regression coefficients (pairwise tests) from the mixed models was obtained using the following steps: (1) constructing a model based on the null hypothesis of no differences between groups (H_0_); (2) resampling with replacement the distribution of the response residuals under the H_0_ model, reconstructing a bootstrap y-response vector, and refitting the H_1_ model to the bootstrap response vectors to generate 10,000 bootstrap estimates of the regression coefficients (*β*) under H_0_; and (3) comparing the observed value of *β* against the H_0_ bootstrap distribution (two-tailed, frequentist *p*-value). Due to the fact that males and females did not significantly differ on any measure of average daily sleep, physical activity or mood (all *P* < 0.05; see section "Results"), we did not include sex as a covariate in the model.

To separately evaluate within (*β*_*W*_) and between-subjects (*β*_*B*_) effects, following standard guidelines^31^, we fit separate regression models for within-participant and between-participant analyses. To evaluate within-participant effects, we first within-participant centered predictor variables by computing within-participant *z*-scores prior to fitting the LMM. To evaluate between-participant effects, the model was fit to participant means. This procedure ensures that within and between-participant effects can be estimated separately and are not confounded^31^. Mixed-model construction and bootstrapping were performed with the lme4 package^48^ in the R environment (R Development Core Team, 2015)^49^. Intervariable correlations were measured using the nonparametric Spearman’s *ρ* test. Corrections for multiple comparisons were performed using the false discovery rate (FDR)^50^.

## Data Availability

The datasets generated during and/or analyzed during the current study are available from the corresponding author on reasonable request.
